# The Impact of Posttraumatic Stress Disorder on Clinical Presentation and Psychosocial Treatment Response in Youth with Functional Abdominal Pain Disorders: An Exploratory Study

**DOI:** 10.3390/children7060056

**Published:** 2020-06-02

**Authors:** Sarah Nelson, Natoshia Cunningham

**Affiliations:** 1 Department of Anesthesiology, Critical Care and Pain Medicine, Boston Children’s Hospital, Boston, MA 02115, USA; 2 Department of Psychiatry, Harvard Medical School, Boston, MA 02115, USA; 3 Department of Family Medicine, Michigan State University, East Lansing, MI 48824, USA; natoshia@msu.edu

**Keywords:** pediatric, functional abdominal pain, posttraumatic stress disorder, cognitive behavioral therapy, treatment response

## Abstract

Youth with functional abdominal pain disorders (FAPDs) may report high rates of trauma and/or posttraumatic stress disorder (PTSD), which could impact both physical and psychosocial functioning, in addition to psychosocial treatment response. The current study aimed to examine the rates of PTSD in a sample of 89 youth with FAPDs and examine the association between PTSD with physical and psychosocial functioning. The impact of PTSD on psychosocial treatment response in a subsample of youth with FAPDs was also explored. Participants were youth with FAPDs (ages 9–14) enrolled in a larger study examining the effect of a short-term pain and anxiety focused cognitive behavioral therapy (CBT) treatment (Aim to Decrease Anxiety and Pain Treatment (ADAPT)) for youth with FAPDs. Youth were administered a semi-structured diagnostic interview by a trained clinician to confirm the presence of psychological diagnoses, including PTSD. Measures of physical and psychosocial functioning were also completed. Results revealed a high rate of PTSD in youth with FAPDs with 12.4% meeting diagnostic criteria for the disorder. PTSD was associated with several indicators of increased psychosocial impairment and one indicator of physical impairment. Exploratory analyses revealed comorbid PTSD may impact response to a brief CBT intervention targeting pain and anxiety, but more rigorous controlled studies are needed.

## 1. Introduction

Youth with chronic pain report higher rates of psychological trauma (abuse, neglect, violent or conflictual home environment, etc.) when compared to the average population or healthy controls [1,2]. In particular, functional abdominal pain disorders (FAPDs), which are common, persistent, and/or recurrent abdominal pain conditions without a clear organic etiology, are worthy of further investigation in relation to trauma. In fact, a history of traumatic events and/or life stressors may lead to the development of persistent abdominal pain in a subset of youth [3,4]. A significant subset of youth with FAPDs also report a high rate of functional disability (e.g., minimizing physical activity) [5,6] and psychological impairment (e.g., anxiety, depression [5,7,8], somatic symptoms [9,10], and distress [11]) that may be related to the high rates of trauma experiences in this group [2]. Despite this, the link between pediatric FAPDs, trauma, and other clinical indicators, such as functional impairment and treatment response, have not yet been investigated.

There is some evidence that the prevalence of FAPDs is significantly higher in adults who report psychological stress and a history of psychological trauma [8,12,13]. However, minimal research has been carried out in pediatric populations. In a notable exception, a recent study performed in a large heterogenous sample of youth with chronic pain presenting for evaluation to a tertiary chronic pain clinic found 83% of youth (up to age 18) who presented with FAPDs reported at least one adverse childhood experience (ACE; e.g., abuse or neglect, parental separation or divorce, violent or conflictual home environment etc.) in their lifetime [2]. Results of this same study indicated that youth with chronic pain (FAPDs and other conditions) and a history of ACEs reported higher rates of anxiety, depression, and fear of pain compared to youth with lower or no history of ACEs [2]. In comparison, epidemiologic studies on trauma in youth indicates that rates can range from 8–12% (sexual assault) to 38–70% (witness to serious community violence) for single incident exposure with 20–48% of youth reporting multiple types of victimization [14]. Evidence in pain populations specifically also suggests that youth with heterogenous forms of chronic pain and a history of trauma are more likely to report symptoms of posttraumatic stress disorder (PTSD; re-experiencing, impacted mood or cognitions, bad dreams about the event, hypervigilance, etc. [15]) when compared to youth without chronic pain, [16] and it has been proposed that PTSD may maintain chronic pain symptoms in youth [17]. However, the rates of PTSD and its impact on functioning have not previously been examined in youth with FAPDs specifically. Nor is it understood how trauma impacts psychological treatment response in youth with chronic pain conditions like FAPDs. This lack of research in youth with FAPDs is striking, given that these are among the most commonly reported chronic pain conditions affecting youth [12].

In general, the presence of psychological distress, such as anxiety or depression, has been shown to contribute to poorer psychosocial and physical treatment outcomes in youth with chronic pain conditions [18,19]. A recent study in youth with chronic pain found trauma history did not negatively impact psychosocial or physical treatment outcomes among youth presenting for intensive (i.e., day treatment) pain-focused rehabilitation [20]. However, this study was limited to participants enrolled in intensive pain rehabilitation who were required to “fail” (i.e., not show sufficient treatment response) traditional outpatient psychotherapy [21,22]. No study to date has examined post-treatment psychosocial or physical functioning in youth with chronic pain and a history of trauma and/or PTSD in an outpatient setting. It is known that, irrespective of pain, youth with a history of trauma and/or PTSD require targeted psychosocial treatment approaches (e.g., trauma-focused cognitive behavioral therapy [23,24]) and many fail to respond to traditional psychotherapies [25]. Therefore, it may be that youth with trauma and/or PTSD and chronic pain are less likely to respond to traditional pain-focused outpatient psychotherapy and would thus benefit from a trauma-focused approach, but this remains unclear.

Aims of the current study included examining (1) the rates of PTSD in youth with FAPDs and (2) the association between PTSD and psychosocial (i.e., anxiety, depression, pain catastrophizing) and physical impairment (i.e., functional disability, pain intensity, somatization) in youth with FAPDs when compared to youth with FAPDs and no PTSD history. Further, we explored the association between PTSD diagnosis and post-treatment functioning (i.e., psychosocial and physical impairment) in youth with FAPDs enrolled in a short-term cognitive behavioral therapy to target pain and comorbid anxiety [26]. To begin, it was hypothesized that a significantly higher rate of youth with FAPDs would meet diagnostic criteria for PTSD at baseline when compared to the average population. This is based on the high rate of ACE exposure in youth with FAPDs (up to 83%) [2], comparatively lower rates of ACEs in general pediatric populations (37–67% [27]), and the previous studies on rates of posttraumatic stress in pediatric pain populations (e.g., up to 32% report posttraumatic stress symptoms [non-diagnostic] [16]) compared to general pediatric populations (ranging between 0.4% and 15.9% [28,29]). Considering the association between PTSD and poorer psychosocial and physical impairment in adult and/or mixed chronic pain populations, it was also hypothesized that youth with FAPDs who meet criteria for PTSD would evidence poorer psychosocial and physical functioning at baseline when compared to youth with FAPDs and no PTSD. It was also hypothesized that PTSD would adversely affect the response to cognitive behavioral therapy (CBT) for pain and anxiety, when compared to youth with FAPDs and no PTSD diagnosis.

## 2. Materials and Methods

### 2.1. Participants

Eligible participants were youth between the ages of 9 and 14 who were part of a larger clinical trial for youth with FAPDs. Among the larger participant sample, youth were included in the current study if they had completed a psychological assessment as part of the larger study and were randomized to one of the two intervention groups (see below). Participants with a history of severe depression or active suicidal ideation were excluded from participating in the larger clinical trial and in the current study.

### 2.2. Procedures and Intervention

Data were gathered as part of a larger study examining outcomes of a stepped care approach to providing a brief CBT intervention—Aim to Decrease Anxiety and Pain Treatment (ADAPT)—for youth with functional abdominal pain disorders (FAPDs). Data were collected in person by a trained clinical research coordinator (bachelor’s level) or trained study clinician (postdoctoral fellow) at a large Midwestern United States children’s hospital. All study procedures were supervised by a licensed clinical psychologist and approved by the hospital Institutional Review Board. All participants consented to the treatment and were required to endorse at least some functional disability due to their pain in order to qualify for entrance into the study (initial screening), in addition to completing measures of pain and anxiety. They then received enhanced usual care, consisting of psychoeducation on pain coping and access to relaxation strategies, during this same visit to their pediatric gastroenterologist. If participants continued to show evidence of impacted functional disability after two weeks (Functional Disability Inventory (FDI) re-screening, completed over the phone), they were scheduled for a comprehensive baseline assessment in person. Then, qualifying participants were randomized to receive either the Aim to Decrease Anxiety and Pain Treatment (ADAPT) intervention or medical treatment as usual (TAU). Participants completed a post-assessment similar in nature to the baseline assessment one week following the completion of ADAPT or TAU. The total enrollment time in the study was approximately 10 weeks.

ADAPT was developed by the senior author (N.C.) drawing from an established CBT protocol for pain management [30] and “Cool Kids” to treat anxiety [31]. ADAPT targets both pain and anxiety symptoms, utilizes a blend of 2 in-person sessions and 4 web modules with interventionist phone support designed to be easily accessible and ensure consistent content delivery for all participants. Both interventions (ADAPT and TAU) took place for a total of 6 weeks. Additional information is available in the ADAPT treatment development paper [26] and the results of the Randomized Clinical Trial (RCT), which are currently under review.

### 2.3. Measures of Participant Functioning

#### 2.3.1. Physical Functioning

##### Pain Intensity

Average pain levels measured with the visual analogue scale (VAS: 0–10) in the past 2 weeks were collected at study baseline and post-treatment [32].

##### Functional Disability Inventory—Child Version

Fifteen-item validated measure of disability in youth with chronic pain [33]. Higher scores indicate greater disability, with scores >7 indicating evidence of at least some (e.g., more than minimal) disability for enrollment in this study. There are established cut-offs for mild (0–12), moderate (13–29), and severe (30+) disability [33]. A change of >7.8 points indicates a clinically significant difference [18]. Internal consistency in the current sample was 0.85 (re-screen) and 0.89 (post-treatment), which is good. The FDI was completed at study screening, re-screening (over the phone), and post-treatment. For the purposes of the current study, only re-screening and post-treatment scores were utilized.

##### Children’s Somatization Inventory (CSI)-25

Assesses the severity of nonspecific somatic symptoms (e.g., “weakness” and “dizziness”) not necessarily linked to organic disease etiology [34]. Participants rated the extent to which they have experienced each of the symptoms during the last 2 weeks using a 5-point Likert scale ranging from 0 = “not at all” to 4 = “a whole lot”. Total scores are computed by summing the items and higher scores indicate higher levels of somatic symptoms. The CSI is found to have adequate reliability and validity [34]. The CSI was collected at baseline and post-treatment. Internal consistency in the current sample was excellent at baseline (α = 0.91) and good (α = 0.89) at post-treatment.

#### 2.3.2. Psychosocial Functioning

##### Pain Catastrophizing Scale (PCS)

The Pain Catastrophizing Scale, child (PCS-C [35]) assesses children’s negative thinking associated with pain. Reponses are rated on a 5-point Likert scale ranging from 0–4 (“Not at all true” to “Very true”). All items are summed to produce a total score with 30 or higher indicative of clinically significant pain catastrophizing [35]. Participants were asked to complete the PCS at baseline and post-treatment. Internal consistency for the PCS-C in the current sample was 0.93 (baseline) and 0.94 (post-treatment), which is excellent.

##### Screen for Child Anxiety-Related Disorders (SCARED)

Validated measure of child anxiety [36,37]. Scores greater than or equal to 25 indicate clinically significant anxiety [36,37]. The SCARED was completed at baseline and post-treatment. Internal consistency in the current sample was 0.94 (baseline) and 0.95 (post-treatment), which is excellent.

##### Children’s Depression Inventory-Second Edition, (CDI-2)

Patient-report measures of children’s depressive symptoms within the past 2 weeks, and an inventory valid for children ages 7 and older [38,39]. Higher scores indicate more depressive symptoms. The CDI was completed at baseline and post-treatment. Internal consistency in the current sample was good (α = 0.89) at baseline and excellent (α = 0.92) at post-treatment.

##### Anxiety Disorder Interview Schedule (ADIS)—Child Version

A psychometrically reliable diagnostic interview [40] conducted under the supervision of a licensed clinical psychologist by either a trained clinician (post-baccalaureate and pursuing graduate study in psychology) or postdoctoral psychology fellow, to assess for childhood psychiatric disorders, including posttraumatic stress disorder (PTSD) in addition to anxiety, mood, obsessive-compulsive, and related disorders such as somatic symptoms or substance use. In addition to going through diagnostic criteria for all other diagnoses, participants were coded as having PTSD for the current study if they endorsed a Category A traumatic event (e.g., abuse, neglect) per Diagnostic and Statistical Manual (DSM-5) criteria [15] and endorsed the required amount of symptomatology with an assigned clinician severity rating to indicate the presence of a clinically significant diagnosis [40]. For all diagnoses, a clinician severity rating was originally assigned by the administering clinician and then reviewed in supervision with a licensed clinical psychologist. The ADIS was administered at baseline and post-treatment.

### 2.4. Statistical Analyses

All statistical analyses were performed using SPSS v. 24 (IBM Corp, Chicago, IL, USA) [41]. To begin, descriptive statistics were computed for age, gender, and race/ethnicity in addition to all primary outcomes across baseline and post-treatment time points. PTSD groups were formulated by dichotomously coding (0, 1) participants by the presence of clinician-verified PTSD symptoms through a semi-structured interview (ADIS). Following this, in order to examine baseline differences in physical and psychosocial impairment by PTSD group (0 = No PTSD; 1 = PTSD), a Mann–Whitney U test was performed with PTSD as the grouping variable (Aim 1). Non-parametric analyses were chosen due to the small sample size of the PTSD group. Dependent variables were all continuous and included psychosocial (i.e., pain catastrophizing (PCS), anxiety (SCARED), and depression (CDI)) and physical (i.e., pain intensity (VAS), functional disability (FDI), somatization (CSI)) indictors (all of which were collected at baseline/Time 1). Additional ADIS diagnoses excluding PTSD were also populated across the sample into a count variable and compared by PTSD group (0, 1) via a Mann–Whitney U test. Treatment response, conceptualized as post-treatment (i.e., Time 2) response, was also explored in youth with FAPDs with and without PTSD using Mann–Whitney U tests. Data were isolated by intervention randomization (ADAPT or TAU) or PTSD group (PTSD only) and then a series of tests were performed within these subsets with either PTSD or intervention randomization as the grouping variable and Time 2 psychosocial and physical functioning as the dependent variables. 

## 3. Results

### 3.1. Participant Demographics

Among those youth who were screened for the larger trial, participants were included in the current study if they completed baseline procedures and were randomized to receive either the ADAPT or TAU intervention. The final sample included 89 participants (59.6% female, 89.9% White or Caucasian), with an average age of 11.73 (SD = 2.15). At baseline, participants met criteria for an average of 2 (M = 2.39) diagnoses assessed via the Anxiety Disorder Interview Schedule (ADIS). Most common diagnoses were generalized anxiety disorder (27%), social anxiety disorder (19.1%), and separation anxiety disorder (16.9%). Participants within the sample via self-report questionnaires also met criteria for clinically significant anxiety (SCARED; mean = 35.08), moderate functional disability (FDI; mean = 20.72), and moderate pain intensity (VAS; mean = 3.8). See Table 1 for complete details on demographics and functioning at baseline across the sample. 

### 3.2. Incidence of PTSD in Youth with FAPDs

Results of the ADIS semi-structured interview revealed that 12.4% (*n* = 11, 90.9% female) of the sample currently met criteria for a current posttraumatic stress disorder (PTSD) diagnosis at baseline. Among these participants, the average clinician severity rating indicates severe impact (mean score: 6.4, range: 4–8; SD: 1.36). All participants with a clinically significant diagnosis of PTSD at baseline remained in the study throughout both time points.

### 3.3. PTSD in Relation to Baseline Physical and Psychosocial Functioning in Youth with FAPDs

At baseline, the results of Mann–Whitney U tests with PTSD as the grouping variable (0, 1) and psychosocial (anxiety, depressive symptoms, pain catastrophizing) and physical (functional disability, somatization, average pain intensity) functioning as the dependent variables revealed that youth with FAPDs and PTSD reported significantly higher rates of anxiety (Z = −2.898, *p* = 0.004), pain catastrophizing (Z = −3.118, *p* = 0.002), and somatization (Z = −2.691, *p* = 0.007) when compared to youth with FAPDs and no PTSD, with functional disability trending towards significance (Z = −1.905, *p* = 0.057). In addition, youth with FAPDs and PTSD reported pain catastrophizing levels (M = 36.91; SD = 8.95) that were clinically significant (>30) when compared to youth with FAPDs and no PTSD, who reported a subclinical average level of pain catastrophizing (M = 25.15; SD = 11.27). When comparing the results of clinician-administered diagnostic interviews (Anxiety Disorder Interview Schedule (ADIS)) across PTSD groups, the results of Mann–Whitney U tests also revealed that youth with PTSD on average evidenced a significantly higher amount of ADIS diagnoses (M = 3.55; SD = 1.45) when compared to youth without PTSD (M = 2.25; SD = 1.70). Table 2 details the mean (M) and standard deviation (SD) of each study variable by PTSD group for the whole sample (across both conditions).

### 3.4. Exploratory Analyses: PTSD in Relation to Psychosocial Treatment Response in Youth with FAPDs

Following baseline assessments, 44 participants (49.4%) were randomized to the ADAPT intervention and 45 participants (50.6%) were randomized to treatment as usual (TAU). Among the PTSD group in particular, 4 (36.4%) were randomized to ADAPT and 7 (63.6%) were randomized to TAU. A total of 79 participants completed the study (*n* = 40 in the ADAPT group). In order to primarily examine the effect that PTSD may have on intervention response, descriptive statistics (mean, standard deviation) for post-treatment (Time 2) measures of impairment were compared (Table 3). Data were then isolated by intervention randomization (ADAPT and then TAU) and Mann–Whitney U tests were performed again in these data subsets with PTSD as the grouping variable and psychosocial and physical variables were included as dependent variables. Results for the ADAPT intervention indicated a significant difference in anxiety (Z = −2.008, *p* = 0.042) and somatization (Z = −2.295, *p* = 0.017) between PTSD groups but not pain catastrophizing, functional disability, depressive symptoms, or pain intensity. Specifically, those with PTSD had higher levels of anxiety and somatization following ADAPT compared to those without PTSD. Results for the TAU intervention indicated no significant differences in any of the psychosocial or physical variables between PTSD groups. After isolating the PTSD group only, results of Mann–Whitney U tests with intervention randomization as the grouping variable (ADAPT versus TAU) revealed that there were no significant differences in any treatment outcomes (all *p*’s > 0.05).

## 4. Discussion

The current study aimed to examine the incidence of posttraumatic stress disorder (PTSD) and its association with physical and psychosocial functioning in youth with functional abdominal pain disorders (FAPDs). The findings are timely given the lack of research on rates of PTSD in pediatric FAPDs, and the extremely limited understanding of how PTSD relates to psychosocial and physical impairment in youth with FAPDs. Moreover, it is currently unknown how psychological intervention outcomes are affected for youth with chronic pain conditions such as FAPDs and PTSD. Therefore, an exploratory aim was also to examine outcomes for youth with FAPDs and PTSD vs. no PTSD who completed a brief pain and anxiety focused cognitive behavioral intervention (Aim to Decrease Anxiety and Pain Treatment (ADAPT)) [26].

Results of the current study indicated that, when assessed via a gold-standard semi-structured clinical interview, 12.4% of youth with FAPDs meet diagnostic criteria for PTSD. On average, their symptoms were rated by the administering clinician as in the “severe” range. In non-pain pediatric populations, epidemiologic studies in youth (under 18) have reported incidence rates of PTSD as low as 0.4% overall, with rates higher in females (0.7%) vs. males (0.1%) [42]. Conversely, a large meta-analysis of studies using a semi-structured interview revealed lifetime rates of PTSD in (non-pain) youth to be as high as 15.9% across both males and females [43]. By comparison, a recent study examining rates of self-reported posttraumatic stress symptoms (PTSS) in a larger heterogenous pediatric pain population found that 32% of youth reported PTSS [16]. Results of the current study are consistent with previous studies of PTSS in pediatric pain samples in that findings suggest PTSS/PTSD rates to be high when compared (with some estimates) to the average population. However, the disparate rates of PTSD/PTSS found across both pediatric pain and population sample studies may be due to the varying methods of screening or diagnostic procedures by study. For example, Noel, et al. utilized a self-report screening questionnaire (Child PTSD symptom checklist-5; CPSS-5 [44,45]), whereas the current study utilized a semi-structured diagnostic interview by a trained clinician to query current diagnosis (ADIS [40]). Both methods are important (e.g., self-report measures can certainly be more practical to administer) and provide a better idea of how these symptoms may manifest in pediatric pain populations, but the use of semi-structured interviews by a trained clinician are considered the gold standard for diagnostic (vs. symptom) assessment of psychological conditions [42,46]. Therefore, future research should extend the use of semi-structured diagnostic interviews to other pediatric pain populations in order to get a more complete idea of how the diagnosis of PTSD may manifest in these youth.

When comparing youth with FAPDs with and without PTSD in the current sample, results revealed that youth with FAPDs and PTSD reported higher baseline rates of impairment in several areas of psychosocial functioning and one aspect of physical functioning. Specifically, higher rates of anxiety, pain catastrophizing, and somatization were observed in youth with PTSD versus no PTSD (*p*’s < 0.05). These results were clinically significant (total score >30 vs. <30) in the case of pain catastrophizing. Depressive symptoms, pain intensity, and functional disability were not statistically or clinically significantly different between PTSD groups, although the functional disability was trending towards significance. These findings are generally consistent with previous research on the effects of psychological trauma and/or PTSD on pediatric or young adult chronic pain populations in that trauma/PTSD history has been most commonly found to affect psychosocial functioning vs. physical functioning [1]. However, previous research on chronic pain and posttraumatic stress symptoms (PTSS) found a strong association between PTSS and facets of the pain experience, including intensity, interference, and unpleasantness [16]. Given that functional disability was trending towards significance in the current sample, it may be that clearer differences in functional impairment between youth with and without PTSD can be found in larger or more diverse pain samples. Future research should continue to examine these relationships. The significant differences in somatic symptoms between youth with and without PTSD are also interesting in that somatization (i.e., somatic symptoms), like FAPD, often does not have a concrete medical explanation [3,6]. In FAPDs in particular, it has been proposed that somatization mediates the relationship between abdominal pain and psychological dysfunction such as anxiety and/or depression [47]. Concomitantly, research indicates that the presence of clinical anxiety symptoms negatively impacts outcomes in youth with FAPDs [7,19,48]. It may be that the presence of PTSD via high somatization increases risk for poorer long-term outcomes in youth with FAPDs, but this has yet to be examined.

Results of exploratory analyses examining treatment response in youth with FAPDs and PTSD vs. no PTSD indicated youth with PTSD enrolled in the ADAPT intervention generally reported significantly higher rates of anxiety and somatization symptoms at post-assessment, although rates of these symptoms were also higher in those with PTSD at baseline. However, given that these differences persist post-treatment, these results are consistent with the larger trauma literature that has evidenced the negative impact that psychological trauma and/or PTSD history can have on psychosocial treatment response [24,25]. As previous research in pediatric pain samples has also found that a significant subset of youth experience decreased response to traditional psychosocial treatment [18,19], it may be that identifying potential risk factors for poorer outcomes such as psychological trauma and/or PTSD history will enhance positive treatment response and potentially increase prevention through the development or use of catered psychosocial interventions (e.g., Trauma-Focused Cognitive Behavioral Therapy [23], Mindfulness-Based Stress Reduction [49], etc.). Perhaps ADAPT could be further enhanced and refined to directly target PTSD that occurs in a large minority of youth with FAPDs. Future research should continue to examine potential avenues for prevention and intervention optimization in pediatric pain populations. Finally, a notable finding was the comparatively low rates of depressive symptoms vs. anxiety in the current sample, both at baseline and post-treatment. These findings are notable given the strong co-occurrence between anxiety and depressive symptoms in youth [50] but consistent with other studies examining psychological impairment in youth with FAPDs [7,8,51]. It may be that youth who are more likely to report anxiety over depression are at an increased risk for somatization and, in turn, abdominal pain. However, this remains unclear. It is also known that abdominal pain in childhood is a risk factor for the development of depressive symptoms over time [52], so early identification and treatment of youth with FAPDs and comorbid anxiety are critical.

The current study has several strengths. To begin, the use of a clinician-administered semi-structured interview is currently the gold standard in the identification of posttraumatic stress disorder (PTSD) [29] and likely minimized bias in reporting of trauma and PTSD [53]. The recruitment and screening of treatment-seeking youth with FAPDs allowed for a nuanced understanding of how trauma impacts this chronic pain condition in youth in particular. The most significant limitations of the current study include the small sample size of youth with PTSD who received active treatment (*n* = 4). This limits interpretability and ability to draw meaningful conclusions from results and likely underpowered analyses. The homogeneity of the participant sample, while consistent with published studies from other pediatric pain samples, also limits the generalizability of results to broader pediatric pain populations. Future research should replicate these analyses in larger samples and include more complex longitudinal examinations (e.g., controlling for baseline functioning) with larger treatment-seeking pain samples with and without PTSD.

## 5. Summary and Conclusions

Results of the current study are the first to examine the incidence of PTSD and its association with poorer functioning in youth with FAPDs. Youth with FAPDs reported higher rates of PTSD than healthy populations, and the presence of PTSD is associated with increases in psychosocial impairment. Further, the exploration of data in a subset of youth with FAPDs and PTSD suggests a negative effect on treatment response, but further investigation is needed. Future research should continue to examine these relationships in larger populations, and with additional pain populations, with the ultimate goal of optimizing treatment for this at-risk pain population. 

## Figures and Tables

**Table 1:** Participant characteristics (N = 89).

	M	SD	Range
**Age**	11.73	2.15	9–14
	**N**	**%**	
**Gender**			
**Female**	53	59.6	
**Male**	36	40.4	
**Race**			
**White**	80	89.9	
**Black**	4	4.5	
**Hispanic or Latino**	2	2.2	
**American Indian or Alaskan Native**	1	1.1	
**Biracial**	1	1.1	
**Other**	1	1.1	
	N	%	
**Posttraumatic Stress Disorder (PTSD**)	11	12.4	
	**M**	**SD**	**Range**
**ADIS Diagnoses**	2.39	1.7	0–6
**Psychosocial Functioning**			
**Anxiety (SCARED)**	35.08	16.5	0–77
**Depressive Symptoms (CDI)**	27.19	2.69	20–36
**Pain Catastrophizing (PCS)**	26.65	11.64	2–52
**Physical Functioning**			
**Functional Disability (FDI)**	20.72	8.89	8–39
**Somatization (CSI)**	32.21	16.24	5–76
**Average Pain Intensity (VAS)**	3.85	1.94	0–8.7

Note: Child Depression Inventory reported in T-scores.

**Table 2:** Variability in variables across PTSD groups (baseline/Time 1).

	PTSD (*n* = 11)		No PTSD (*n* = 78)			
	M	SD	M	SD	z	*p*
**Psychosocial Functioning**						
Anxiety	47.63	12.29	33.24	16.31	−2.898	0.004
Depressive Symptoms	28.18	2.52	27.05	2.71	−1.326	0.185
Pain Catastrophizing	36.91	8.95	25.15	11.27	−3.118	0.002
**Physical Functioning**						
Functional Disability	25.9	9.65	19.97	8.59	−1.905	0.057
Somatization	46.09	18.26	30.17	14.99	−2.691	0.007
Average Pain Intensity	4.27	1.87	3.79	1.95	−0.874	0.382

**Table 3:** Mean post-treatment (Time 2) outcome scores by PTSD group and intervention randomization (Aim to Decrease Anxiety and Pain Treatment (ADAPT) vs. treatment as usual (TAU)).

	Anxiety	Depressive Symptoms	Pain Catastrophizing	Functional Disability	Somatization	Average Pain Intensity
	Mean (SD)	Mean (SD)	Mean (SD)	Mean (SD)	Mean (SD)	Mean (SD)
PTSD ^a^-ADAPT ^b^	39.00 ^^^ (17.45)	26.50 (2.52)	19.50 (14.06)	17.00 (12.52)	39.50 ^^^ (16.66)	2.64 (1.05)
PTSD-TAU ^c^	32.67 (14.84)	26.17 (2.79)	22.67 (13.29)	9.83 (4.62)	26.67 (12.77)	1.58 (1.76)
noPTSD ^d^-ADAPT	20.54 ^^^ (15.41)	26.82 (2.12)	14.60 (9.02)	9.40 (8.44)	20.29 ^^^ (12.75)	2.44 (2.19)
noPTSD-TAU	27.38 (15.92)	27.81 (2.58)	22.47 (13.51)	12.09 (9.58)	23.50 (12.87)	3.08 (2.22)

^^^ Results are significantly different (*p* < 0.05) between youth with and without PTSD who completed ADAPT. No other significant differences were found between PTSD groups or participants enrolled in the TAU intervention. ^a^ Youth with a baseline history of posttraumatic stress disorder (PTSD); ^b^ Aim to Decrease Anxiety and Pain Treatment (ADAPT); ^c^ Treatment as usual (TAU); ^d^ Youth without a baseline history of posttraumatic stress disorder.

## References

[1] Nelson S., Cunningham N., Peugh J., Jagpal A., Arnold L.M., Lynch-Jordan A., Kashikar-Zuck S. (2017). Clinical profiles of young adults with juvenile-onset fibromyalgia with and without a history of trauma. Arthritis Care Res..

[2] Nelson S., Simons L., Logan D. (2018). The Incidence of Adverse Childhood Experiences (ACEs) and their Association with Pain-related and Psychosocial Impairment in Youth with Chronic Pain. Clin. J. Pain.

[3] Korterink J.J., Diederen K., Benninga M.A., Tabbers M.M. (2015). Epidemiology of pediatric functional abdominal pain disorders: A meta-analysis. PLoS ONE.

[4] Afari N., Ahumada S.M., Wright L.J., Mostoufi S., Golnari G., Reis V., Cuneo J.G. (2014). Psychological trauma and functional somatic syndromes: A systematic review and meta-analysis. Psychosom. Med..

[5] Cunningham N.R., Lynch-Jordan A., Barnett K., Peugh J., Sil S., Goldschneider K., Kashikar-Zuck S. (2014). Child pain catastrophizing mediates the relation between parent responses to pain and disability in youth with functional abdominal pain. J. Pediatr. Gastroenterol. Nutr..

[6] Walker L.S., Sherman A.L., Bruehl S., Garber J., Smith C.A. (2012). Functional abdominal pain patient subtypes in childhood predict functional gastrointestinal disorders with chronic pain and psychiatric comorbidities in adolescence and adulthood. Pain.

[7] Cunningham N.R., Lynch-Jordan A., Mezoff A.G., Farrell M.K., Cohen M.B., Kashikar-Zuck S. (2013). Importance of addressing anxiety in youth with functional abdominal pain: Suggested guidelines for physicians. J. Pediatr. Gastroenterol. Nutr..

[8] Campo J.V., Bridge J., Ehmann M., Altman S., Lucas A., Birmaher B., Di Lorenzo C., Iyengar S., Brent D.A. (2004). Recurrent abdominal pain, anxiety, and depression in primary care. Pediatrics.

[9] Little C.A., Williams S.E., Puzanovova M., Rudzinski E.R., Walker L.S. (2007). Multiple somatic symptoms linked to positive screen for depression in pediatric patients with chronic abdominal pain. J. Pediatr. Gastroenterol. Nutr..

[10] Walker L.S., Garber J., Smith C.A., Van Slyke D.A., Claar R.L. (2001). The relation of daily stressors to somatic and emotional symptoms in children with and without recurrent abdominal pain. J. Consult. Clin. Psychol..

[11] Love S.C., Mara C.A., Kalomiris A.E., Cunningham N.R. (2019). The Influence of Caregiver Distress and Child Anxiety in Predicting Child Somatization in Youth with Functional Abdominal Pain Disorders. Children.

[12] Rasquin A., Di Lorenzo C., Forbes D., Guiraldes E., Hyams J.S., Staiano A., Walker L.S. (2006). Childhood functional gastrointestinal disorders: Child/adolescent. Gastroenterology.

[13] Devanarayana N., Rajindrajith S., Karunanayake A., Nishanthini S., Perera M.S., Benninga M.A. (2012). Abdominal pain predominant functional gastrointestinal diseases: Association with child abuse, traumatic life events and quality of life. J. Gastroenterol. Hepatol..

[14] Saunders B.E., Adams Z.W. (2014). Epidemiology of traumatic experiences in childhood. Child Adolesc. Psychiatr. Clin. N Am..

[15] Association A.P. (2013). Diagnostic and Statistical Manual of Mental Disorders (DSM-5®).

[16] Noel M., Wilson A.C., Holley A.L., Durkin L., Patton M., Palermo T.M. (2016). Posttraumatic stress disorder symptoms in youth with vs. without chronic pain. Pain.

[17] Holley A.L., Wilson A.C., Noel M., Palermo T.M. (2016). Post-traumatic stress symptoms in children and adolescents with chronic pain: A topical review of the literature and a proposed framework for future research. Eur. J. Pain.

[18] Sil S., Arnold L.M., Lynch-Jordan A., Ting T.V., Peugh J., Cunningham N., Powers S.W., Lovell D.J., Hashkes P.J., Passo M. (2014). Identifying treatment responders and predictors of improvement after cognitive-behavioral therapy for juvenile fibromyalgia. Pain.

[19] Cunningham N.R., Jagpal A., Tran S.T., Kashikar-Zuck S., Goldschneider K.R., Coghill R.C., Lynch-Jordan A.M. (2016). Anxiety adversely impacts response to cognitive behavioral therapy in children with chronic pain. J. Pediatr..

[20] Nelson S., Smith K., Sethna N., Logan D. (2019). Youth With Chronic Pain and a History of Adverse Childhood Experiences in the Context of Multidisciplinary Pain Rehabilitation. Clin. J. Pain.

[21] Logan D.E., Carpino E.A., Chiang G., Condon M., Firn E., Gaughan V.J. (2012). A day-hospital approach to treatment of pediatric complex regional pain syndrome: Initial functional outcomes. Clin. J. Pain.

[22] Simons L.E., Sieberg C.B., Pielech M., Conroy C., Logan D.E. (2013). What does it take? Comparing intensive rehabilitation to outpatient treatment for children with significant pain-related disability. J Pediatr Psychol.

[23] Mannarino A.P., Cohen J.A., Deblinger E. (2014). Trauma-Focused Cognitive-Behavioral Therapy. Evidence-Based Approaches for the Treatment of Maltreated Children.

[24] Barbe R.P., Bridge J.A., Birmaher B., Kolko D.J., Brent D.A. (2004). Lifetime history of sexual abuse, clinical presentation, and outcome in a clinical trial for adolescent depression. J. Clin. Psychiatry.

[25] Shamseddeen W., Asarnow J.R., Clarke G., Vitiello B., Wagner K.D., Birmaher B., Keller M.B., Emslie G., Iyengar S., Ryan N.D. (2011). Impact of physical and sexual abuse on treatment response in the Treatment of Resistant Depression in Adolescent Study (TORDIA). J. Am. Acad. Child Adolesc. Psychiatry.

[26] Cunningham N.R., Nelson S., Jagpal A., Moorman E., Farrell M., Pentiuk S., Kashikar-Zuck S. (2018). Development of the Aim to Decrease Anxiety and Pain Treatment for Pediatric Functional Abdominal Pain Disorders. J. Pediatr. Gastroenterol. Nutr..

[27] Garrido E.F., Weiler L.M., Taussig H.N. (2018). Adverse Childhood Experiences and Health-Risk Behaviors in Vulnerable Early Adolescents. J. Early Adolesc..

[28] Hamblen J., Barnett E. (2016). PTSD in Children and Adolescents. www.ncptsd.org.

[29] Wilson J.P., Keane T.M. (2004). Assessing Psychological Trauma and PTSD.

[30] Kashikar-Zuck S., Ting T.V., Arnold L.M., Bean J., Powers S.W., Graham T.B., Passo M.H., Schikler K.N., Hashkes P.J., Spalding S. (2012). Cognitive behavioral therapy for the treatment of juvenile fibromyalgia: A multisite, single-blind, randomized, controlled clinical trial. Arthritis Rheum..

[31] Lyneham H., Abbott M., Wignall A., Rapee R. (2003). The Cool Kids Anxiety Treatment Program.

[32] Price D.D., Bush F.M., Long S., Harkins S.W. (1994). A comparison of pain measurement characteristics of mechanical visual analogue and simple numerical rating scales. Pain.

[33] Kashikar-Zuck S., Flowers S.R., Claar R.L., Guite J.W., Logan D.E., Lynch-Jordan A.M., Palermod T.M., Wilson A.C. (2011). Clinical utility and validity of the Functional Disability Inventory among a multicenter sample of youth with chronic pain. Pain.

[34] Walker L.S., Beck J.E., Garber J., Lambert W. (2008). Children’s Somatization Inventory: Psychometric properties of the revised form (CSI-24). J. Pediatr. Psychol..

[35] Pielech M., Ryan M., Logan D., Kaczynski K., White M.T., Simons L.E. (2014). Pain catastrophizing in children with chronic pain and their parents: Proposed clinical reference points and reexamination of the Pain Catastrophizing Scale measure. Pain.

[36] Cunningham N.R., Jagpal A., Nelson S., Jastrowski Mano K.E., Tran S.T., Lynch-Jordan A.M., Hainsworth K., Peugh J., Mara C., Kashikar-Zuck S. (2019). Clinical Reference Points for the Screen for Child Anxiety-related Disorders in 2 Investigations of Youth With Chronic Pain. Clin. J. Pain.

[37] Jastrowski Mano K.E., Evans J.R., Tran S.T., Anderson Khan K., Weisman S.J., Hainsworth K.R. (2012). The psychometric properties of the screen for child anxiety related emotional disorders in pediatric chronic pain. J. Pediatr. Psychol..

[38] Kovacs M. (2014). Children’s Depression Inventory (CDI and CDI 2). Encycl. Clin. Psychol..

[39] Logan D.E., Claar R.L., Guite J.W., Kashikar-Zuck S., Lynch-Jordan A., Palermo T.M., Wilson A.C., Zhou C. (2013). Factor structure of the children’s depression inventory in a multisite sample of children and adolescents with chronic pain. J. Pain.

[40] Silverman W.K., Albano A.M. (1996). Anxiety Disorders Interview Schedule: Adis-IV Child Interview Schedule.

[41] Wagner W.E. (2019). Using IBM® SPSS® Statistics for Research Methods and Social Science Statistics.

[42] Kearney C.A., Wechsler A., Kaur H., Lemos-Miller A. (2010). Posttraumatic stress disorder in maltreated youth: A review of contemporary research and thought. Clin. Child Fam. Psychol. Rev..

[43] Alisic E., Zalta A.K., Van Wesel F., Larsen S.E., Hafstad G.S., Hassanpour K., Smid G.E. (2014). Rates of post-traumatic stress disorder in trauma-exposed children and adolescents: Meta-analysis. Br. J. Psychiatry.

[44] Foa E.B., Johnson K.M., Feeny N.C., Treadwell K.R. (2001). The Child PTSD Symptom Scale: A preliminary examination of its psychometric properties. J. Clin. Child Psychol..

[45] Foa E.B., Asnaani A., Zang Y., Capaldi S., Yeh R. (2018). Psychometrics of the Child PTSD Symptom Scale for DSM-5 for trauma-exposed children and adolescents. J. Clin. Child Adolesc. Psychol..

[46] Gerson R., Rappaport N. (2013). Traumatic stress and posttraumatic stress disorder in youth: Recent research findings on clinical impact, assessment, and treatment. J. Adolesc. Health.

[47] Lavigne J.V., Saps M., Bryant F.B. (2013). Models of anxiety, depression, somatization, and coping as predictors of abdominal pain in a community sample of school-age children. J. Pediatr. Psychol..

[48] Cunningham N.R., Jagpal A., Peugh J., Farrell M.K., Cohen M.B., Mezoff A.G., Lynch-Jordan A., Kashikar-Zuck S. (2017). Risk Categorization Predicts Disability in Pain-associated Functional Gastrointestinal Disorders After 6 Months. J. Pediatr. Gastroenterol. Nutr..

[49] Rosenzweig S., Greeson J.M., Reibel D.K., Green J.S., Jasser S.A., Beasley D. (2010). Mindfulness-based stress reduction for chronic pain conditions: Variation in treatment outcomes and role of home meditation practice. J. Psychosom. Res..

[50] Starr L.R., Davila J. (2012). Responding to anxiety with rumination and hopelessness: Mechanism of anxiety-depression symptom co-occurrence?. Cogn. Ther. Res..

[51] Youssef N.N., Atienza K., Langseder A.L., Strauss R.S. (2008). Chronic abdominal pain and depressive symptoms: Analysis of the national longitudinal study of adolescent health. Clin. Gastroenterol. Hepatol..

[52] Shelby G.D., Shirkey K.C., Sherman A.L., Beck J.E., Haman K., Shears A.R., Horst S.N., Smith C.A., Garber J., Walker L.S. (2013). Functional abdominal pain in childhood and long-term vulnerability to anxiety disorders. Pediatrics.

[53] Hardt J., Rutter M. (2004). Validity of adult retrospective reports of adverse childhood experiences: Review of the evidence. J. Child Psychol. Psychiatry.

